# Supplementary material for: Possibilities of Implementing Hospital-Based Health Technology Assessment (HB-HTA) at the Level of Voivodeship Offices in Poland
Source: Int J Environ Res Public Health. 2022 Sep 7;19(18):11235. doi: 10.3390/ijerph191811235 (PMC9517551; doi:10.3390/ijerph191811235)
Supplement: Supplementary file 1 [file ijerph-19-11235-s001.zip › ijerph-1815156-supplementary.pdf]

Table S1. Questions answered by an interviewess.

1. What do you mean by medical innovation?
a. Does the Polish health care system have any developed instruments,<br>a mechanism for implementing innovations pol. healthcare facilities?<br>b. What do we consider to be the biggest barrier to the implementation of innovations?
2. What are the benefits of implementing HB HTA from the perspective of:
a. Your institution?<br>b. healthcare system?<br>c. hospital?
3. What role can your institution play in implementing HB HTA?<br>What barriers to this involvement do you see?
4. In the proposed model of implementation of HB HTA, such roles were assigned institutions like: Voivodeship offices, NHF, AOTMiT, MoH. Which of these institutions should have the greatest impact on the implementation of HB HTA?
5. Are there any other institutions in the healthcare system that could play a significant role in the implementation of HB-HTA? If so, what are these?
6. The novelty in the planned HB HTA implementation model is the Department of assignment of a special role to the Health of the Voivodeship Offices, which is to review the reports of HB HTA prepared in hospitals:
a. Are the Health Department of Voivodeship Offices prepared / capable of fulfilling this new role? If not, what should be done to improve it?<br>b. Should reports be provided free of charge?<br>c. Should there be a fee for reviews?
7. What barriers do you see in implementing the HB HTA?
8. How can themselves motivate hospitals to implement HB HTA?
9. What negative phenomena, in your opinion, may appear after the implementation of HB-HTA?
